# Exploration of Surgeon Motivations in Management of Abdominal Wall Hernias: A Qualitative Study

**DOI:** 10.1001/jamanetworkopen.2020.15916

**Published:** 2020-09-15

**Authors:** C. Ann Vitous, Sara M. Jafri, Claire Seven, Anne P. Ehlers, Michael J. Englesbe, Justin Dimick, Dana A. Telem

**Affiliations:** 1 Center for Healthcare Outcomes and Policy, University of Michigan, Ann Arbor; 2 Department of Surgery, University of Michigan, Ann Arbor

## Abstract

**Importance:**

Although evidence-based guidelines designed to minimize health care variation and promote effective care are widely accepted, creating guidelines alone does not often lead to the desired practice change. Such knowledge-to-practice gaps are well-recognized in the management of patients with abdominal wall hernia, where wide variation in patient selection and operative approach likely contributes to suboptimal patient outcomes. To create sustainable, scalable, and widespread adherence to evidence-based guidelines, it is imperative to better understand individual surgeon motivations and behaviors associated with surgical decision-making.

**Objective:**

To evaluate the systematic application of the Theoretical Domains Framework (TDF) to explore motivations and behaviors associated with surgical decision-making in abdominal wall hernia practice to help inform the future design of theory-based interventions for desired practice and behavior change.

**Design, Setting, and Participants:**

This qualitative study used purposive sampling to recruit 21 practicing surgeons at community and academic hospitals from 5 health regions across Michigan. It used interviews consisting of clinical vignettes for highly controversial situations in abdominal wall hernia repair, followed by semistructured interview questions based on the domains of the TDF to gain nuance into motivating factors associated with surgical practice. Patterns within the data were located, analyzed, and identified through thematic analysis using software. All data were collected between May and July 2018, and data analysis was performed from August 2018 to July 2019.

**Main Outcomes and Measures:**

Factors associated with decisions on the surgical approach to abdominal wall hernia repair were assessed using TDF.

**Results:**

Seventeen (81%) of the 21 participants were men, with a median (interquartile range) age of 47 (45-54) years. Of the 14 TDF domains, 5 were found to be most associated with decisions on the surgical approach to abdominal wall hernia repair for surgeons in Michigan: knowledge, beliefs about consequences, social or professional role and identity, environmental context and resources, and social influences. Mapping of the findings to the sources of behavior identified the potential intervention functions and policy categories that could be targeted for intervention. The intervention functions found to be most relevant included education, persuasion, modeling, incentivization, and environmental restructuring.

**Conclusions and Relevance:**

Using the TDF, this study found that the primary factors associated with individual practice were opinion leaders, practice conformity, and reputational concerns. These findings are important because they challenge traditional dogma, which relies mainly on dissemination of published evidence, education, and technical skills acquisition to achieve evidence-based practice. Such knowledge allows for the development of sustainable, theory-based interventions for adherence to evidence-based guidelines.

## Key Points

**Question:**

How can implementation frameworks such as the Theoretical Domains Framework be used to identify domains associated with the adoption of best practices in surgery?

**Findings:**

This qualitative study used abdominal wall hernia as a case study and found that the surgeon knowledge, beliefs about the consequences, social or professional role and identity, environmental context and resources, and social influences domains were associated with decision-making.

**Meaning:**

These findings suggest that implementation frameworks offer a method for better understanding which factors motivate behavior change among surgeons.

## Teaser

This qualitative study evaluates the systematic application of the Theoretical Domains Framework to explore motivations and behaviors associated with surgical decision-making in abdominal wall hernia practice.

## Introduction

Variation in health care contributing to poor patient outcomes is well documented.^1,2,3,4^ Although evidence-based guidelines designed to minimize health care variation and promote effective care are widely available, publishing a guideline alone often does not create the desired practice change.^5,6,7,8,9,10^ Health interventions based on evidence-based guidelines commonly fail to produce the intended effect when introduced into practice. Recommendations are often disseminated without consideration of either the behavior change necessary to promote sustainable adoption or the individual and organizational barriers preventing implementation.^11,12,13,14^ Understanding how to strengthen adherence to evidence-based guidelines is a critical knowledge gap impacting the care of surgical patients.

Abdominal wall hernia is a key example of a common, but morbid, condition where wide variation in surgical approach exists. Evidence-based guidelines pertaining to both patient selection and elements of operative technique are available, although often not heeded.^15,16,17,18,19,20,21,22^ For example, a recent population-based study^17,18,19,20,21,22,23^ found that up to 25% of persons undergoing elective abdominal wall hernia repair are actively smoking or morbidly obese or have inadequate glycemic control. Furthermore, data also demonstrate underutilization of minimally invasive techniques in patients who may benefit from this approach.^15,16,17^ These data demonstrate deviation from evidence-based guidelines, which is associated with suboptimal patient outcomes and increased episodes of care payments resulting from readmissions, recurrences, and complications for persons undergoing abdominal wall hernia repair.^24,25,26^ The underlying causes of variation in adoption of evidence-based hernia guidelines are unknown, with the motivations and behaviors associated with individual surgeon practice largely unexplored. Consequently, the optimal mechanism for widespread, sustainable implementation of evidence-based guidelines remains unknown.

In this context, we sought to qualitatively explore factors associated with surgeon decision-making for abdominal wall hernia repair that cannot be adequately captured through a quantitative approach. Through the systematic use of the Theoretical Domains Framework (TDF), we aimed to understand the dominant factors informing surgeon behavior.

## Methods

The study protocol was approved by the University of Michigan Medicine institutional review board. All participants were provided with an oral informed consent statement and verbally consented before their interview. This qualitative study follows the Consolidated Criteria for Reporting Qualitative Research (COREQ) reporting guideline.

### Study Design

A variety of frameworks, models, and theories exist to guide implementation efforts. We focus on the TDF, given its ties to evidence-based behavior change techniques and because it addresses changes that need to occur at the individual level, such as decisions for surgery.^27,28,29^ Using the TDF to explore modifiable individual behaviors associated with surgeon decision-making helps select appropriate evidence-based behavior change techniques and identifies evidence-based strategies to implement tailored, evidence-based hernia recommendations. Although we recognize that factors beyond the individual (eg, organizational or regional) are associated with differential outcomes, the TDF was specifically selected because it best matches our study design, which focuses on the individual hernia surgeon, and because it allows for mapping of TDF domains to evidence-based behavior change techniques (Figure).

**Figure.**
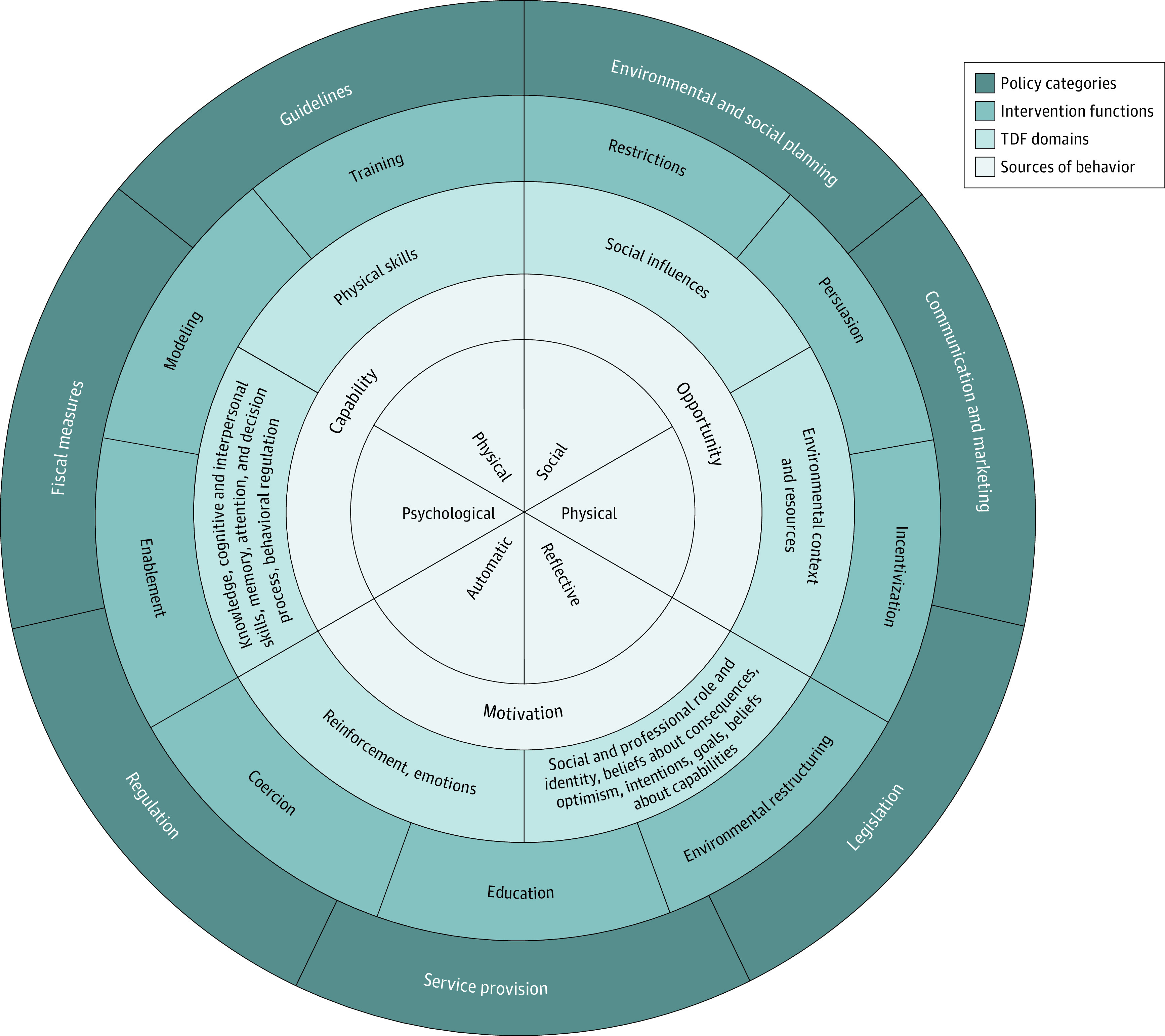
Behavior Wheel TDF indicates Theoretical Domains Framework.

The TDF includes 14 domains and 84 constructs covering the physical and social environment, as well as individual motivation and capability factors.^22^ The domains include the following: (1) knowledge; (2) skills; (3) social or professional role and identity; (4) beliefs about capabilities; (5) optimism; (6) beliefs about consequences; (7) reinforcement; (8) intentions; (9) goals; (10) memory, attention, and decision processes; (11) environmental context; (12) social influences; (13) emotion; and (14) behavioral regulation.^28^ Although, to our knowledge, no formal guidance exists on applying the TDF, we selected an approach proposed by Atkins and colleagues.^30^

### Study Population

We used purposive sampling to identify study participants from the Michigan Surgical Quality Collaborative, which represents 70 community, tertiary care, and academic sites across the state of Michigan.^31^ All participants were required to be practicing surgeons who perform abdominal wall hernia repairs. Twenty-one participants were recruited from 8 community and 13 academic hospitals. Participants were diverse with respect to age, years in practice, and hospital setting and reflected the current demographic characteristics of surgeons in Michigan (Table 1).

**Table 1.** Participant Demographic, Training, and Practice Characteristics

Category	Participants, No. (%) (N = 21)
Gender	
Male	17 (81)
Female	4 (19)
Age, y	
35-44	9 (43)
45-54	8 (38)
55-64	3 (14)
≥65	1 (5)
Race	
White	18 (86)
Prefer not to answer	3 (14)
Ethnicity	
Non-Hispanic or non-Latino	16 (76)
Middle Eastern	1 (5)
No response given	4 (19)
Degree	
MD	14 (67)
DO	7 (33)
Time in practice, y	
0-4	2 (9)
5-10	5 (24)
11-15	5 (24)
16-20	3 (14)
≥21	6 (29)
Completed fellowship training	
Yes	6 (29)
No	15 (71)
Fellowship concentration	
Trauma	2 (33)
Minimally invasive	3 (50)
Surgical critical care	1 (17)
Hospital demographic	
Community	8 (38)
Academic	13 (62)
Self-reported percentage of cases involving abdominal wall hernia repair	
11-24	14 (67)
25-49	4 (19)
50-74	3 (14)

### Interview Topic Guide

The interview guide included clinical vignettes pertaining to controversial situations in abdominal wall hernia repair, followed by questions mapping to each TDF domain (Table 2). Interview questions explored physician-based variation and factors associated with decision-making behavior in hernia surgery, whereas the clinical vignettes provided nuance into those processes. We sought to determine factors associated with variation based on patient factors (eg, body mass index, tobacco use, the role of glycemic control, and age) and hernia factors (eg, size, location, and symptoms). Two pilot interviews were completed with surgeons who met study eligibility criteria, informing slight modifications made to the subsequent guide. Because only minor changes were made, initial interviews were included in the analysis.

**Table 2.** Crosswalk for Draft Interview Guide to the Theoretical Domains Framework

Theoretical Domains Framework domain	Questions
Knowledge	In your medical opinion, what is the evidence for using MIS in hernia repair?
	What guidelines do you follow for hernia repair? Can you describe the process of how you develop and implement those guidelines?
Skills	Describe your personal experience in treating patients with hernias.
	Can you describe any formal or informal training that your received in MIS?
Social or professional role and identity	How would you describe the consistency in approaches to hernia repairs among surgeons in your practice?
Beliefs about capabilities	In what ways, if any, do your feelings influence whether or how you use MIS?
Optimism	In your medical opinion, how will treating an index patient with MIS impact the patient in the short-term? What about the long-term?
Beliefs about consequences	In your medical opinion, what are the benefits, if any, of using MIS vs open repair?
	If you sensed that not using MIS damaged your relationships in any way (with patients or physicians), would this change the way that you thought about it?
	In your experience, what are the perceptions of how patients feel after MIS vs open approaches?
Reinforcement	How important is it to you to have MIS as part of your hernia practice?
	If you sensed that not using MIS damaged your relationships in any way (with patients or physicians), would this change the way that you thought about it?
Intentions	How many patients do you anticipate treating with MIS over the next year? What about with open repair?
Goals	How important is it to you to have MIS as part of your hernia practice?
Memory, attention and decision processes	Walk me through the steps you take in your decision to approach a hernia with minimally invasive technique vs an open technique.
Environmental context and resources	What are the main barriers to you using MIS? What about the facilitators?
Social influences	What influential individuals or groups are in favor or against using MIS? Can you describe their perspective on MIS?
	How do the opinions of these people influence your decisions on whether or how you use MIS for hernia repair?
Emotion	In what ways, if any, do your feelings influence whether or how you use MIS?
Behavioral regulation	What are the main barriers to you using MIS? What about the facilitators?

Abbreviation: MIS, minimally invasive surgery.

### Data Collection

An email was sent to all potential participants identified through the Michigan Surgical Quality Collaborative outlining the purpose of the project and requesting their participation in a survey designed to gather demographic characteristics. Of the 31 surgeons who completed the survey, 21 agreed to participate in interviews. Reasons for nonparticipation were not elicited. Independent interviews were conducted by 3 authors (C.A.V., S.M.J., and S.V.), a qualitative analyst and 2 research assistants, with extensive experience in interviewing surgeons by telephone or in person. Interviews were digitally recorded and lasted between 30 and 60 minutes. All recordings were transcribed and deidentified. Observations about each interview (ie, field notes) were documented after each interview. Interviews continued until data saturation was reached, which was determined when new themes emerged infrequently, and the code definitions remained stable.^32^ All data were collected between May and July 2018. Transcripts were not returned to participants for review.

### Statistical Analysis

After data collection, we began iteratively analyzing the data, incorporating both deductive and inductive components in our thematic analysis. The deductive components included creating codes based on queries mapped to each TDF domain. From there, we used inductive analysis to differentiate the constructs in relation to the domains.

Three members of the research team (C.A.V., S.M.J., and S.V.) created the initial codebook by reviewing transcripts and meeting to collate ideas. Once the initial codebook was agreed upon, 2 members (C.A.V. and S.M.J.) independently coded each transcript using NVivo data analysis software version 11.4.3 (QSR International), blinded to the other’s work. In areas where text could be categorized under multiple domains, it was coded where it fit best. In situations where consensus could not be reached, an implementation science expert (D.A.T.) provided guidance.

After data coding, we generated reports for all codes under each domain. All members of the research team met regularly to reach consensus on relevant domains. The main factors that were considered concurrently for determining domain relevancy included frequency of specific participant beliefs or themes, presence of conflicting beliefs, and evidence of strong beliefs that may be associated with target behavior.^30^ For example, if a substantial number of participants reported having an average or above-average skill set with minimally invasive surgery (MIS), skill set was not considered a factor associated with surgical approach. In contrast, if a substantial number of participants cited access to resources as a barrier or facilitator, it was considered a domain associated with surgical approach. A descriptive matrix was used to synthesize all relevant responses.^33^ Data analysis was performed from August 2018 to July 2019.

## Results

Seventeen (81%) of the 21 participants were men, with a median (interquartile range) age of 47 (45-54) years. Key themes emerging from the interviews with surgeons were organized within 5 TDF domains: knowledge, beliefs about consequences, social or professional role and identity, environmental context and resources, and social influences. The domains were further broken down into relevant constructs, including identity; professional identity, boundaries, or role; organizational culture or climate; material resources; social support; group conformity; social pressure; knowledge; scientific rationale; and outcome expectancies (Table 3).

**Table 3.** Representative Quotations

Domains	Constructs	Quotations	Participants, No.
Social or professional role and identity: coherent set of behaviors and displayed personal qualities of an individual in a social or work setting	Identity	“I mean, I’m an MIS surgeon, so sure. Ask a pizza man what’s for dinner, you know, you get similar.”	6
		“So, when you adopt a new technology, there’s always a learning curve. And that if someone who’s been repairing hernias open for 25 years starts doing them with a different technique, are they doing to have more complications? Probably, yes. So, it makes it harder to adopt new technology, and I think from their perspective, if it ain’t broke, don’t fix it.”	
	Professional identity, boundaries, or role	“We have one surgeon who’s probably the busiest that only does mesh plugs and he won’t do it any other way. He doesn’t do laparoscopic stuff… But he’s the most senior surgeon in our community and there’s no, you know, from a market standpoint, there’s no reason for him to change, because people just get referred to him.”	11
		“My one partner does no laparoscopics, no robotic hernia repairs. And if he sees somebody he thinks that we’d be better at, he sends it to one of us to do. I decided not to do robotic so that my younger partner could be better at it, you know, because I’m not always going to be around forever.”	
		“I don’t have that here simply because the other surgical group in town, the other group of general surgeons, most of them aren’t really doing the kind of repairs that my partner and I are doing. So, because the information is out there in our community, a lot of those patients are just coming to us anyway.”	
Environmental context and resources: any circumstance of a person’s situation or environment that discourages or encourages the development of skills and abilities, independence, social competence, and adaptive behavior	Organizational culture or climate	“Bigger institutions that have programs that have multiple people that are working in there and residents and things like that, I think it’s a little bit easier to introduce some of that stuff because there is a variability that’s expected at a larger institution where you have the ability to say, hey, this is the latest greatest, and this is how we’re going to use it.”	13
		“There’s always a lot of politics with minimally invasive or with the robot, and at least up here, I mean, with the hospital and who gets to do it, who’s privileged to do it. And it’s driven by a lot of other forces than just patient care unfortunately.”	
	Material resources	“Some factors are sometimes an issue like, you know, if let’s say there’s block time available but not in one of the MIS rooms, like we only have certain rooms with robotic equipment or with high-definition towers for laparoscopies and, you know, I’ve had a patient say, well, I’d just rather have the hernia repair done a week sooner, and you can just do it open. I would say, okay.”	17
		“And then number two is, at our hospital, we have difficult-to-do robotic cases that really have a lack of assistance, because when you do a robotic case, you’re going to sit at the console away from the patient to do the surgery, and someone needs to stand at the bedside. And that person standing at the bedside, we lack those people.”	
		“For emergent or urgent cases, quite honestly, that alone might define sometimes whether it’s minimally invasive or not. So, on a weekend, I have a team that’s comfortable setting up for me to do a minimally invasive, and it’s a good candidate, I’ll do it. If my team looks at me like I’ve just asked them to parade a unicorn down the OR hallway, then I know that that patient’s probably going to get a delay, and we’re not going to get it done for them, and then I might end up doing it open because it’s more rigmarole and more difficult to convince the team to do it with me.”	
		“I mean, you know, like, of course, you would think, yeah, that’s obvious. But, you know, in the real world, this is how we make a living. So, you know, sometimes it’s a hard decision to say, okay, like I’m not going to, I’m going to give my job away. I’m going to give my money away because I just want, I want the patient to have the latest and greatest repair, basically.”	
Social influences: interpersonal processes that can cause individuals to change their thoughts, feelings, or behaviors	Social support	“Having peers who are experienced, you know, ask for help, being trained in it as a, you know, being younger and being trained in it, having fellowship training, sometimes courses or opportunity to retool and relearn.”	5
		“I don’t think they necessarily influence my opinion to use it. I think my opinion comes specifically more from, you know, my experience with how patients have done and the research. But they definitely gave kind of guided, you know, a potentially better, a better way to perform an MIS technique and kind of more just reassured that, you know, the MIS technique is probably the proper way to do it.”	
	Group conformity	“But for the run-of-the-mill hernias, inguinal hernia, my partners are now mostly doing them robotically too, but that’s a newer trend in my group. For a long time, they were doing them all open… So that’s a recent change in our practice.”	6
		“I would say, in our hernia, in our advanced hernia clinic, we’re all pretty consistent. We all pretty much follow the same guidelines that I mentioned, and we’re all pretty consistent on what other approaches that we do. In my general office, we do have a little more inconsistency.”	
	Social pressure	“Oh, yeah, if the patient would prefer to have an open procedure, then I would do that. If the patient would prefer to have a laparoscopic approach, I would do that. But I tell them what my concerns are with that approach and why I was leaning more toward the other way. And if they are still dogmatic about it, I’ll do it their way, and, you know, just be cautionary with them that we may have to convert to the other way if.”	7
		“But the public wants whatever is new. And they want it without any knowledge of whether it’s you know, before we know if it’s better or worse. And I still don’t think we know if it’s better or worse.”	
		“But I’ve got a lot of partners that say, oh, you should try it, because it’s cool to use it. And, you know, I don’t, I’m not going to do that, you know. But if they can show me evidence that it’s better, sure, I’ll think about it.”	
Knowledge: awareness of the existence of something	Knowledge	“Well, the majority of it is, you know, by far, is just, you know, my experience, and over the years.”	21
		“And some of it is experience-based, but you can’t always go by just your experience. You have to, it’s more effective to use the literature. So, I am heavily evidence-based.”	
		“The Hernia Society has had a big influence in the kind of the, like the International Hernia Collaboration, which is an online group, I think there’s a lot more consensus then there was 10, 15 years ago, but it’s still, there’s still not broad consensus overall.”	
		“Yeah, so there’s, you can find evidence for anything you want to do, right? So, there are always going to be people who use evidence to support their own practice.”	
		“I’ve seen some pretty robust guys, you know, argue one way or the other without having the literature behind them, and it’s meaningless to do such.”	
		“And so, it’s bad to say this, but I think that also a lot of times it depends on who’s doing what research. Because at one time you’ll have, you know, one of the recognized top hernia experts in the world talking about one particular repair, and it’s because at that moment in time they’re getting funded by somebody to do that research. And then a month later when the next person comes up and says, hey, how about this? Then they’re going to change gears and they’re going to be talking about how this is the best group here.”	
		“For the long term, I think we need more research. I think we need to be able to follow that long term… But that’s, it’s just kind of a physiologic, you know, created in your head, yeah, this looks better to me kind of thing. And it’s not necessarily scientifically based.”	
		“But minimally invasive versus open approach for long term outcome is really what we’re looking at now. I don’t think the data is out there for that yet.”	
	Scientific rationale	“I guess I’ve never used any official guidelines like from the Hernia Societies. I just always had my own guidelines, I guess, based on my experience of doing a lot of these hernias.”	8
		“But I just, like I ask it in my templates when somebody comes in for a hernia consult, like I ask all these questions, and if any of the diabetic stuff, obesity stuff, like constipation, like those red flags always target, you know, a specific strategy before doing surgery. But I can’t, I’m sure that there’s literature on, everything I do has got to have some kind of rhyme or reason. So, what I’ve developed over the years is usually evidence-based in some way.”	
		“It’s a little bit of the Wild West, I feel like. So right now, there’s a ton of practice variation, even within my small group… And so right now, I’m sort of cherry picking between the things that seem to have the highest, the greatest concordance.”	
Beliefs about consequences: acceptance of the truth, reality, or validity about outcomes of a behavior in a given situation	Outcome expectancies	“I mean, I think it’s the normal stuff. It’s a little less painful. Recovery is a little better. It’s more cosmetic, less wound infections, the usual.”	18
		“Like you’d think the minimally invasive approach would have less pain, but it doesn’t. It hurts just as much.”	
		“I think all of the studies have shown return to work, return to, you know, activities of daily living are better with laparoscopic approach. The minimally invasive approach is less painful, you know. So, less narcotics, less complications related to narcotics, particularly less opioid-induced constipation, so the reduction in narcotics is a big deal.”	
		“I think, particularly, for a laparoscopic inguinal extra peritoneal, there’s excellent evidence that the reoccurrence rate is extremely low and the infection rate is extremely low.”	
		“Long term, I guess I really don’t perceive any difference in the two.”	
		“I think MIS, or, I mean, I think it’s good for recurrence. I think it’s really good for recurrence. I think it gives you better anatomy, I mean, you can see the anatomy better.”	
		“Typically do most hernias robotically, though I don’t really think there’s a huge difference, with robotic being open.”	
		“I think it’s kind of a tossup, you know. Everybody out at eight weeks looks about the same, whether you do it open or laparoscopic.”	

Abbreviation: MIS, minimally invasive surgery; OR, operating room.

### Social or Professional Role and Identity

For some participants, technology adoption was connected to personal identity. For example, surgeons who received MIS training expressed a bias toward MIS approaches. Conversely, surgeons not trained in MIS expressed hesitancy in changing their approach because of the steep learning curve involved. This was particularly true for middle-to-late career surgeons, because learning a new approach had the potential to increase complications, potentially jeopardizing their reputation and patient care.

Practice patterns reflected professional boundaries and role within a practice or community for some surgeons. This informal process was based on an understanding between surgeons in the community. Participants also described how some surgeons were sufficiently engaged with open repairs, leaving no reason to pursue training in MIS. Finally, in some instances, participants mentioned how surgical groups within their community had reputations for specializing in certain techniques, and patients were typically referred in the best-suited direction.

### Environmental Context or Resources

Participants described how organizational culture was associated with surgical decision-making. Some of these considerations were at an institutional level. For example, participants described how hospital volume could determine the types of cases to which a surgeon had access, with higher-volume hospitals having more exposure to variation in both patients and types of cases. Such variation contributed to providing more preparation on particular techniques and approaches. Organizational climate also was associated with approach selection. For example, participants described how hospital politics influenced who had access to MIS instruments. Furthermore, some participants asserted that in higher-volume hospitals, surgeons had more agency to decline patients who were not ideal surgical candidates. This was reportedly more difficult to implement in lower-volume or community-based practice settings, where surgeons described the tension in turning patients away because they were unable to offer more technically advanced options. This tension was created by the perception that if participants chose not to operate, some patients would seek care elsewhere.

The practice environment was associated with the material resources that were available. For example, scheduling block time was a commonly cited barrier to MIS, with smaller hospitals typically having fewer resources available. Although acquiring additional resources was possible, it often required surgeons to make strong justifications to the administration, with the decision largely dependent on the hospital’s preference. Finally, some participants described how nonphysician team members’ skill set was associated with the approach used. For example, in emergent situations, it was common to have support staff from other specialties, who may be unfamiliar with MIS instruments. In these cases, it was easier for surgeons to convert to an open approach rather than to brief the team members.

### Social Influence

Participants described the role of social support in their decision-making processes. Although few surgeons asserted being entirely uninfluenced by peers, others regularly sought out advice, particularly for difficult cases. It should be noted that although some mentioned considering their peers’ advice, many participants ultimately conceded to objective evidence, such as experiential or published outcomes.

Group conformity also was associated with approach and technique adoption. For example, if a surgeon joined a practice that specialized in MIS, they would adopt that practice, regardless of previous background or training. Similarly, if the majority of a practice’s surgeons were transitioning toward robotics, participants would be inclined to adopting those techniques to avoid being the only group member not offering that approach. Finally, participants suggested that conformity of practices exists more in advanced hernia clinics than in general practice settings.

Social pressure from both patients and colleagues was associated with approach selection. For example, some participants stated that if a patient preferred one approach over another, the surgeon would often concede to the patient’s wishes. In addition, some surgeons made exceptions to evidence-based practices because of the complexity of their relationship with patients. This was more prevalent in smaller communities where surgeons and patients knew each other more personally, leading to difficulty in denying care.

### Knowledge

Knowledge about practice guidelines derived from a combination of factors, including experience, colleagues, and evidence and guidelines. For those who had not received specialized training, participants learned most of their skills from practice partners or colleagues. Participants asserted that knowledge gained early in their careers was instrumental in increasing comfort and exposure to approaches not focused on during training.

Although surgeons asserted the importance of evidence-based practices, some questioned the integrity of the data. Furthermore, although participants cited hernia experts producing important work, some suggested that the source of their research funding might bias what approach they promoted, contributing to the lack of consensus on best practices. Finally, although not the most common response, some surgeons stressed the importance of evidence-based guidelines, stating that it was the determining factor informing their decisions.

In lieu of a lack of consensus with research, surgeons described how they composed their own scientific rationale to determine surgical approach, often based on a combination of experience, patient factors, and anecdotes. Some asserted that these templates were uniquely evidence based and described how they selected elements from various sources that coincided the most.

### Beliefs About Consequences

When reflecting on how outcome expectancies were associated with their approach, many participants asserted a belief in the short-term benefits of MIS over those of an open approach, including a decreased length of stay, quicker recovery, reduced pain, and reduced risk for infection. Long-term benefits, such as decreased risk of recurrence, however, were not viewed with as much certainty, with only a few participants expressing belief in the advantages of MIS over an open approach. Although participants asserted that they were aware of evidence-based guidelines supporting a decreased risk of recurrence with MIS, most stated that the data were not robust enough to support this claim and that 8 weeks after the operation, patients look virtually the same, with the exception of cosmesis. Finally, although it was not the dominant opinion, some participants reported that there were no direct benefits, short- or long-term, of using MIS over an open approach.

### Domains Reported Not Relevant

Eight theoretical domains were less relevant in determining motivations in practice patterns. These were reinforcement, beliefs about capabilities, skills, emotion, goals, optimism, intentions, behavioral regulation, and memory, attention, and decision process.

## Discussion

In this study, the 5 domains most frequently associated with motivating practice patterns for abdominal wall hernia management were knowledge, beliefs about consequences, social or professional role and identity, environmental context and resources, and social influences. In the past, interventions deployed to increase the adoption of evidence-based guidelines in abdominal wall hernia repair focused on factors assumed to be barriers to adoption, such as development of skill set, rather than what was uncovered through the collection of evidence. By incorporating the TDF, we were able to prevent overlooking factors that may be important determinants of practice. By mapping the most frequent domains to the behavior wheel, we identified the sources of behavior, intervention functions, and policy categories that could be targeted for effective intervention (Table 4).

**Table 4.** Identified Barriers and Theoretical Domains Framework Domains Mapped to Evidence-Based Behavior Change

Barrier attribute	Theoretical Domains Framework domains	Source of behavior	Intervention functions	Theory-based strategy
Financial	Beliefs about consequences	Motivation	Environmental restructuring	Prehabilitation program
		Reflective	Education	
Organizational	Environmental context and resources	Opportunity	Incentivization	Payment models (pay for performance)
		Physical	Persuasion	
Practice conformity	Knowledge	Capability	Enabling	Onsite facilitation
		Psychological	Modeling	Payment models
	Social influences	Opportunity	Restrictions	Prehabilitation program
		Social	Persuasion	
	Social or professional role and identity	Motivation	Environmental restructuring	
		Automatic or reflective	Education	
			Coercion	

Effectively encouraging behavior change in clinical settings has been a major challenge.^34^ Although a certain amount of variation is inherent in the management of surgical hernia repair, opportunities exist to tailor care according to patient classification. In areas where evidence-based guidelines exist, there is often a low adoption of surgeons incorporating those guidelines into practice. For example, in recent work exploring the role of sex as a biological variable for abdominal wall hernia repair, Jafri and colleagues^35^ found that despite having practice guidelines addressing hernia in female patients of childbearing age, wide variation in surgeon decision-making exists. With inconsistency being associated with factors such as risk aversion, patient preference, and it simply not being considered, this research emphasizes the need to establish and standardize sex-specific factors through consensus guidelines. Understanding the barriers and facilitators surgeons face in adopting evidence-based guidelines will help in increasing the rates of adoption. Furthermore, in areas where evidence-based guidelines are lacking, understanding motivations associated with surgical decision-making is important for informing the development and implementation of evidence-based guidelines moving forward.

Our data offer further insight into some of these challenges and suggest that any intervention designed to change practice patterns would need to be multifaceted and context specific. Our data expand on research^36^ that suggests the difficulty in identifying high-quality literature to incorporate into practice, calling for the need to develop and disseminate new methods, such as the use of surgical coaching as a platform for communicating ideas. Furthermore, our research expands on literature^37,38^ suggesting that in environments where physicians feel adequately compensated, financial incentives are often ineffective, with little evidence supporting improvement in health care. However, financial motivators are crucial where less security is available, suggesting the need for tailored interventions. Although few participants in our study asserted being strongly motivated by the financial aspects of practice, several cited implicit concerns for not adopting an approach, such as lost referrals and the potential for reputational damages if they started offering approaches that would require them to overcome a steep learning curve. Finally, in environments where developing skill sets would be appropriate, it is imperative to determine avenues that enable surgeons to develop these skills without the fear of it impacting career trajectories.

Significant variability exists in surgical practice. Although resources have been dedicated to address this, the results tend to be both mixed and unpredictable. We demonstrate how TDF utilization can better explain individual surgeon motivations and behaviors associated with surgical decision-making. This study reveals that opinion leaders, practice conformity, and reputational concerns are some of the most important factors associated with individual practice among surgeons performing hernia repair in Michigan. Our findings are key because they challenge current dogma, which has traditionally relied on dissemination of published evidence-based guidelines, education, and skills acquisition to achieve practice change.

### Limitations

There are several limitations to consider when using TDF. First, despite its extensive use in implementation science, there is a lack of formal guidance on how to apply this framework in health care settings.^30^ Until guidelines for the use of TDF are established, there is a need for access to an implementation science expert to provide guidance at many pivotal points in the research process. Next, the process of analyzing the data is time- and labor-intensive, requiring prolonged commitment of research personnel and resources. Additionally, there is a level of subjectivity involved in combining research evidence and matrix mapping to the domains, making it important to have access to adequate guidance, expertise, and resources throughout the research process. Furthermore, although this study allows for understanding of the key determinants associated with decision-making for abdominal wall hernias for surgeons practicing in the state of Michigan, these findings may not be generalizable to other practice settings or environments.

## Conclusions

The design of TDF as a conceptual aid rather than a rigid prescription allows for flexibility, not only in data collection methods, but also in ensuring that the intervention phase can be targeted and based on the identified needs of the specific setting. TDF preserves the focus on behavior change at the individual level, while allowing for consideration of certain aspects of the physical and social environment, establishing a strategy for deciding where to effectively invest time and resources.^30^
